# Endoscopic Ultrasound-Guided Fine-Needle Biopsy Versus Aspiration for Tissue Sampling Adequacy for Molecular Testing in Pancreatic Ductal Adenocarcinoma

**DOI:** 10.3390/cancers16040761

**Published:** 2024-02-12

**Authors:** Wael T. Mohamed, Vinay Jahagirdar, Fouad Jaber, Mohamed K. Ahmed, Ifrah Fatima, Thomas Bierman, Zhuxuan Fu, Philip G. Jones, Amira F. Hassan, Erin Faber, Wendell K. Clarkston, Hassan Ghoz, Ossama W. Tawfik, Sreeni Jonnalagadda

**Affiliations:** 1 Department of Transplant Hepatology, Cleveland Clinic, Cleveland, OH 44114, USA; 2 Department of Internal Medicine, University of Missouri-Kansas City, Kansas City, MO 64108, USA; vinayjaha@gmail.com (V.J.); ifrahfatima@umkc.edu (I.F.); 3 Department of Gastroenterology, University of Missouri-Kansas City, Kansas City, MO 64108, USA; m.ahmed@umkc.edu (M.K.A.); clarkstonw@umkc.edu (W.K.C.); hassanghoz@gmail.com (H.G.); 4 Department of Cardiovascular Research, Saint Luke’s Health System, Kansas City, MO 64108, USA; zfu@saint-lukes.org (Z.F.); pgjones@saint-lukes.org (P.G.J.); 5 Department of Pathology, University of Missouri-Kansas City, Kansas City, MO 64108, USA; amirahassan905@gmail.com (A.F.H.); efaber@mawdpathology.com (E.F.); 6 MAWD Pathology Group, Lenexa, KS 66215, USA; otawfik@mawdpathology.com; 7 Department of Gastroenterology, Saint Luke’s Health System of Kansas City, Kansas City, MO 64108, USA; sjonnalagadda@saint-lukes.org; 8 Department of Pathology, Saint Luke’s Health System of Kansas City, Kansas City, MO 64108, USA

**Keywords:** pancreatic ductal adenocarcinoma (PDAC), endoscopic ultrasound (EUS), fine-needle aspiration (FNA), fine-needle biopsy (FNB), molecular testing

## Abstract

**Simple Summary:**

There is limited literature on sample adequacy for molecular testing and genomic sequencing for pancreatic ductal adenocarcinoma obtained via EUS fine-needle aspiration (FNA) versus EUS fine-needle biopsy (FNB). Thus, we aimed to compare these two modalities regarding sample adequacy for molecular testing and genomic sequencing. In this retrospective chart review study, we analyzed all patients with pancreatic ductal adenocarcinoma who underwent EUS at Saint Luke’s Hospital, Kansas City, MO, from 1 January 2018, to 31 December 2021. A rapid on-site evaluation was performed for all cases by cytotechnologists. We found that out of 1417 EUS procedures, 132 patients underwent EUS-guided biopsies. The mean number of passes required for FNB and FNA was similar. However, EUS-FNB, under rapid on-site specimen evaluation guidance, was superior to FNA in obtaining adequate samples for molecular testing. In addition, tumor surface area and tumor cellularity are essential parameters in determining sample adequacy for molecular testing, regardless of the tissue acquisition modality.

**Abstract:**

Background and Aims: There is limited literature on sample adequacy for molecular testing in pancreatic ductal adenocarcinoma obtained via endoscopic ultrasound (EUS) fine-needle aspiration (FNA) versus EUS fine-needle biopsy (FNB). We aimed to compare these two modalities regarding sample adequacy for molecular and genomic sequencing. Methods: We reviewed all patients with pancreatic ductal adenocarcinoma who underwent EUS at Saint Luke’s Hospital from 2018 to 2021. The patients were categorized based on the method of EUS tissue acquisition, specifically FNA or FNB. A comprehensive evaluation was conducted for all cases by cytotechnologists. Results: Out of 132 patients who underwent EUS-guided biopsies, 76 opted for FNA, 48 opted for FNB, and 8 opted for a combination of both. The average number of passes required for FNB and FNA was 2.58 ± 1.06 and 2.49 ± 1.07, respectively (*p* = 0.704), indicating no significant difference. Interestingly, 71.4% (35) of FNB-obtained samples were deemed adequate for molecular testing, surpassing the 32.1% (26) adequacy observed with FNA (*p* < 0.001). Additionally, 46.4% (26) of FNB-obtained samples were considered adequate for genomic testing, a notable improvement over the 23.8% (20) adequacy observed with FNA (*p* = 0.005). Conclusion: Although the number of passes required for cytologic diagnosis did not differ significantly between EUS-FNB and EUS-FNA, the former demonstrated superiority in obtaining samples adequate for molecular testing. Tumor surface area and cellularity were crucial parameters in determining sample adequacy for molecular testing, irrespective of the chosen tissue acquisition modality.

## 1. Introduction

Pancreatic ductal adenocarcinoma (PDAC) is the third most common cause of cancer death in the United States, with a 5-year survival rate of around 9% [1,2]. Longer median survival times have been reported in patients diagnosed earlier, incidentally, or at operable stages [3,4,5]. Pancreatic masses are generally identified by computerized tomography (CT) of the abdomen. Tissue acquisition is needed for histopathological diagnosis. The standard for obtaining tissue material has shifted from surgical to minimally invasive techniques using endoscopic ultrasound (EUS) guidance [6]. EUS is better at identifying sub-centimeter pancreatic cysts than CT and MRI [7,8]. EUS tissue acquisition (EUS-TA) methods include fine-needle aspiration (FNA) and fine-needle biopsy (FNB). Fine-needle aspiration involves the use of a 22- to 25-gauge needle to obtain a specimen via the excursion of the needle into the lesion. Cellular material is aspirated into the needle core and is subsequently collected in a media solution for processing [9]. Core needle biopsy, on the other hand, employs a slightly larger, hollow-core needle and leverages its cutting action to procure an architecturally preserved tissue core that can be used to make a tissue block for histological analysis. Studies have shown contradictory results regarding the diagnostic yield and adequacy of tissue sampling of FNA vs. FNB [6,10,11].

A meta-analysis of 33 studies and 2984 patients by Hewitt et al. reported that the sensitivity and specificity of EUS-guided FNA (EUS-FNA) for pancreatic neoplasms were 85% and 98%, respectively [12]. Low cellularity and poor histological architecture, which contribute to limited tissue analysis and the poor evaluation of molecular markers, are the major drawbacks of EUS-FNA [13]. EUS-FNB yields more tissue as the needles pick up larger quantities of tissue while preserving the histological architecture. It can be used when FNA is non-diagnostic for detecting molecular markers, for tissue profiling, and for cell culture for targeted therapies [14]. Some studies have shown that EUS-FNB significantly reduces the number of passes required to obtain adequate tissue samples for diagnosis [15,16].

The recent expanding field of precision medicine in oncology has increased the demand for evaluating various molecular markers to determine targeted therapy. Since most malignancies are now commonly diagnosed via minimally invasive techniques, such as FNB and FNA, optimizing tissue acquisition procedures to ensure specimen adequacy of these volume-limited samples for molecular testing is critical. 

Overall, there is adequate but conflicting data on the diagnostic yield of FNA vs. FNB for tissue diagnosis. However, there is limited existing literature on the adequacy of various methods of tissue acquisition for genomic sequencing and molecular testing [17]. Molecular testing of pancreatic adenocarcinomas using next-generation sequencing panels and other procedures is now extensively utilized to implement tailored treatment. The primary objective of our study was to compare EUS-FNA and EUS-FNB in terms of sample adequacy for molecular testing. The secondary outcomes of this study included a comparative analysis of the two modalities in terms of tumor cellularity, tumor surface area, and the number of passes required for successful acquisition.

## 2. Methods

### 2.1. Patient Population

A retrospective chart review was conducted for all patients who underwent EUS-guided tissue biopsy of solid pancreatic masses at Saint Luke’s Hospital (SLH), KC, MO, between 1 January 2018, and 31 December 2021, for PDAC. The Institutional Review Board at Saint Luke’s Hospital Health System approved this study (SLHS-22-007). 

### 2.2. Study Endpoints

The primary aim was to compare EUS-FNA and EUS-FNB modalities in terms of sample adequacy for molecular testing. Sample adequacy was defined as a sample that includes sufficient tumor material based on the requirements of a molecular lab to perform all molecular tests successfully. Our secondary outcomes included conducting a comparative analysis of the two modalities, considering factors such as tumor cellularity, tumor surface area, the number of passes necessary for successful acquisition, procedure complications, and serum CEA levels. 

### 2.3. Procedures Details

All EUS-TA-guided procedures were performed using ROSE for adequacy and for a preliminary diagnosis. The EUS procedure utilized an electronic curvilinear array, either GF-UC140P-AL5 or GF-UTC 180 from Olympus America Inc., Center Valley, PA. Tissue sampling was conducted using commercially available needles. FNB was performed with a 19-, 22-, or 25-gauge Sharkcore needle, whereas FNA was performed with a 19-, 22-, or 25-gauge FNA needle manufactured by Cook Medical, Boston Scientific, or Olympus Endoscopy. Standard suction (dry suction) was applied using a syringe. 

**Figure 1:**
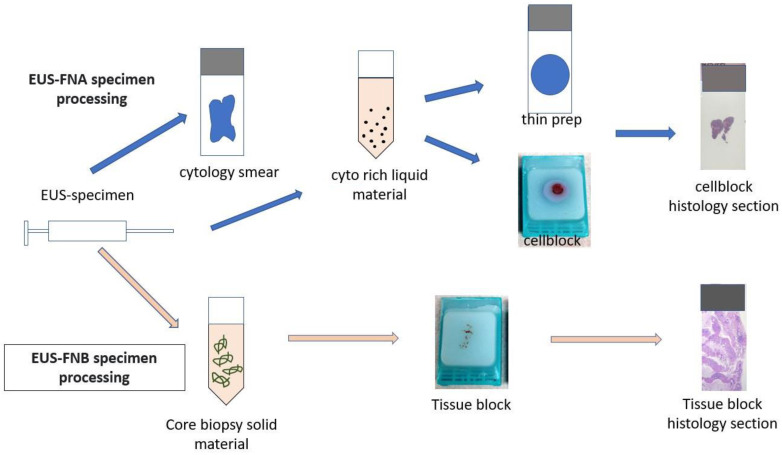
Flowchart of EUS-FNA vs. FNB specimen processing.

**Figure 2:**
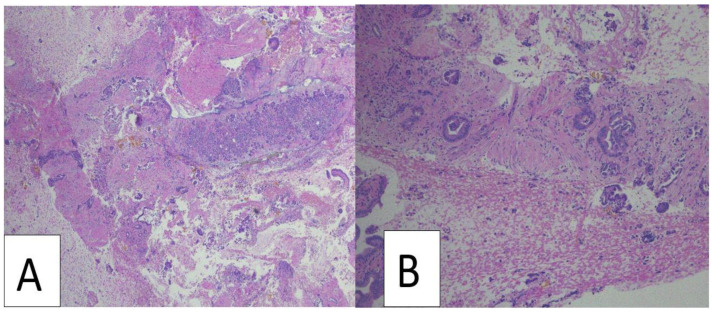
Representative examples of fine needle biopsy samples showing adequate amounts of tissue material with abundant tumor cells suitable for molecular testing ((**A**,**B**), hematoxylin and eosin staining at 40 and 100 magnification, respectively).

For FNA procedures, direct smears were stained using both Diff-Quick and rapid Papanicolaou staining. The FNA rinse was collected in RPMI medium and centrifuged at 1500 RPM for 10 min to prepare cellblock pellets. In patients undergoing FNB, an additional FNA was performed for four patients. Similarly, four patients who underwent FNA first had subsequent sampling performed by FNB. Both cellblocks and FNBs were subsequently fixed in 10% neutral buffered formalin and embedded in paraffin to generate formalin-fixed paraffin-embedded blocks [18] (Figure 1). Hematoxylin and eosin-stained sections were generated for diagnosis and estimation of tumor fraction for additional molecular testing when required. The retained tissue in the cellblock and FNB tissue blocks was preserved for additional testing if needed. Figure 1: Flowchart of EUS-FNA and CNB specimen processing. Figure 2 shows representative examples of FNB samples showing an adequate amount of tissue material with abundant tumor cells suitable for molecular testing. A malignant diagnosis was determined if the pathology report confirmed a diagnosis of PDAC. The reports that mentioned atypical cells or cells suspicious of cancer were excluded from the study. A retrospective slide review of all the cases was performed by a board-certified cytopathologist (OT) to determine the median tumor surface area, tumor cellularity, and specimen adequacy for cytologic diagnosis, immunohistochemical analysis, and molecular testing. Tumor cellularity was divided into <20%, 20–49%, and >49% for each specimen. Tumor cellularity >20% was considered adequate. This definition of the tissue adequacy assessment has been demonstrated to accurately predict successful molecular analysis [19]. Tumor surface area was defined as the area of the slide occupied by tumor cells. It was calculated by demarcating the tumor cells on the slide (excluding blood and non-tumor cells) and calculating the area of the tumor.

Rapid On-Site Evaluation (ROSE) was conducted for all the cases by cytotechnologists. The ROSE procedure involved swift staining and the creation of direct smear slides. No touch imprint cytology was performed in this study. The cytopathology staff reviewed the slides using either on-site microscopy or off-site telepathology with real-time communication with the proceduralist [20]. The final pathological diagnoses and assessment of the tumor surface area, tumor cellularity, and specimen adequacy for molecular testing were performed by board-certified cytopathologists through the examination of FNA and FNB slides. Upon confirming the adequacy of the obtained sample, the sample was sent for next-generation sequencing (NGS). The NGS process encompassed both DNA and RNA tests, the selection of which was determined by factors such as tumor volume and the specific requests of the oncologist. Additionally, as part of our routine practice, MMR immunohistochemistry of the core biopsies was routinely conducted to further enhance the diagnostic evaluation.

### 2.4. Data Collection

We obtained baseline data, including patient demographics, the EUS report procedure details as documented by the endoscopist (including the location, needle gauge size, number of passes, and final pathological diagnosis), and the genomic testing results, through electronic medical record review. 

We recorded clinical parameters, including age, gender, tumor size, location, serum CA 19-9 level, and CEA level, along with technical parameters, such as needle gauge size and the number of EUS procedural passes. 

### 2.5. Statistical Analysis 

The baseline patient characteristics were compared between patients who underwent FNA only, FNB only, or both FNA and FNB. The baseline biopsy characteristics were compared between the FNB and FNA groups. The continuous variables were summarized using the mean ± standard deviation and compared using Student’s T-tests, or were summarized using the median (Q1, Q3) and compared using Wilcoxon tank-sum tests, as appropriate. The categorical variables were summarized using the frequency and percentage and compared using Chi-square or Fisher’s exact tests, as appropriate. 

The Least Square Mean method, adjusted for age, sex, pancreatic lesion location, and size, was used to compare the number of passes between the biopsy technique groups. Multivariate logistic regression adjusted for the same covariates was used to compare the adequacy of tissue sampling for molecular testing and the performance of genomic testing between the biopsy technique groups. The analyses were performed using SAS software (version 9.4). A *p*-value <0.05 was considered statistically significant.

## 3. Results 

Out of 1417 EUS procedures, 132 patients with PDAC underwent EUS-guided biopsies. The patients were stratified based on the mode of tissue acquisition (FNA vs. FNB). This included 76 FNAs, 48 FNBs, and eight combined procedures (Table 1). The percentage of male patients was 45.8% for CNB, 47.4% for FNA, and 75% for combined FNA and CNB cases. Table 1 shows that 94.6% (125/132) of all the evaluated lesions were ≥2 cm^2^ in size, whereas 92.9% of the CNB lesions and 95.2% of the FNA lesions were ≥2 cm^2^. The most common site was the head and uncinate process in the FNB (50.0%) and FNA (59.5%) groups. The second most common site was the body, seen in 12.5% and 13.1% of the lesions in the FNB and FNA groups, respectively. The 22G needle was used most frequently for 58.2% of patients in the FNB group and 82.1% of patients in the FNA group, and the SharkCore^TM^ needle biopsy was the most commonly used needle (Table 1). 

### 3.1. Primary Outcome

**Figure 3:**
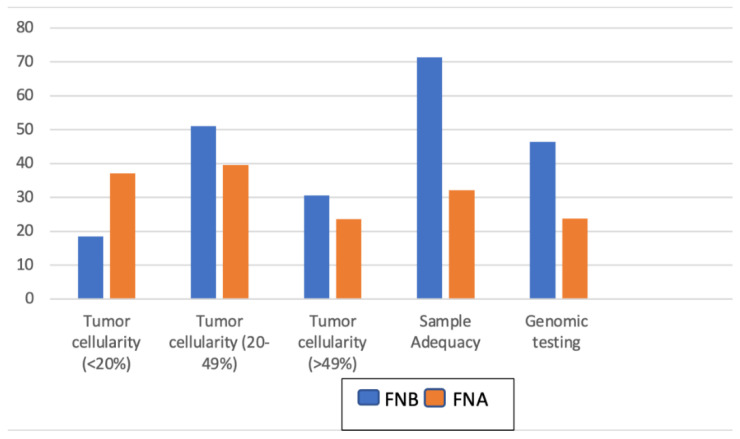
Pathological characteristics of FNA compared to FNB.

An adequate sample for molecular testing was obtained from 71.4% (35) of patients via FNB and 32.1% (26) via FNA (Table 2, Figure 3). Using multivariable logistic regression adjusting for age, sex, pancreatic lesion location, and lesion size, the odds ratio for obtaining an adequate sample using FNB compared to FNA was 5.10 (95% CI 2.27–11.46, *p* < 0.001). Genomic testing of the obtained samples was performed for 46.4% (26/53) of patients in the FNB group and 23.8% (20/84) patients in the FNA group. Using multivariable logistic regression adjusting for age, sex, pancreatic lesion location, and lesion size, the odds ratio for genomic testing performed using FNB compared to FNA was 3.02 (95% CI 1.40–6.53, *p* = 0.005). The mean number of passes was 2.58 ± 1.06 and 2.49 ± 1.07 in the FNB and FNA groups, respectively (Table 2), which was not statistically significant after adjusting for age, sex, pancreatic lesion location, and lesion size (*p* = 0.51).

### 3.2. Secondary Outcomes

Data regarding tumor characteristics were missing in seven cases in the FNB group and three cases in the FNA group. There were significantly larger volumes of tumors collected in the FNB group compared to the FNA group. The median tumor surface area for the CNB group was 25.00 mm^2^ compared to just 4.00 mm^2^ for the FNA group (*p* < 0.001). Similarly, tumor cellularity between 20% and 49% was significantly higher in the CNB group, at 51.0% (25/49), compared to 39.5% (32/81) in the FNA group (*p* = 0.08) (Table 3, Figure 3). The mean number of slides required was 4.30 ± 2.82 in the FNB group and 4.49 ± 2.80 in the FNA group (*p* = 0.70).

The serum CEA level was measured in 15 patients who underwent FNB, with a median of 5.40, 27 patients who underwent FNA, with a median of 5.10, and 2 patients who underwent both modalities, with a median level of 8.70. The serum CA 19-9 level was measured in 35 patients who underwent FNB, with a median of 952, 53 patients who underwent FNA, with a median of 312, and 5 patients who underwent both modalities, with a median of 195. There was no correlation between CEA, CA 19-9, and diagnostic accuracy. No post-procedural complications were reported for any of the patients.

## 4. Discussion

EUS plays a central role in the diagnosis and staging of pancreatic cancer. With the increasing importance of biomarkers for obtaining prognostic information and guiding tailored therapy, the demand for obtaining genomic profiles for pancreatic cancer will continue to grow [15]. The key finding from this study is that FNB is more likely to yield sufficient tissue sampling for molecular and genomic testing compared to FNA. 

Individualizing patient treatment is a core objective of the medical enterprise. Recent advances in molecular science have offered an unprecedented opportunity to achieve this goal through personalized medicine [21]. Realizing the potential benefits of personalized medicine has been elusive, owing to the complex factors contributing to challenges in diagnosing tumors, selecting the appropriate molecular targets, and delivering such complex new services [22]. Within personalized medicine, the availability of targeted therapies in the field of oncology has rapidly evolved from a single locus analysis to broad molecular profiling [23]. Tumor profiling is now the standard of care for many advanced cancers as it identifies the biomarkers that drive therapy selection for the best outcomes [24,25]. Histopathology continues to play a critical role in personalized oncology care [26,27,28]. EUS-guided tissue sampling has emerged as an indispensable method for assessing lesions within the alimentary tract, particularly the pancreas. EUS-guided FNA has long been the gold standard for pancreatic tissue acquisition because of its safety, convenience, and good sensitivity and specificity [29,30]. Unfortunately, these minimally invasive specimens are often inadequate, and the neoplastic material can be depleted as a part of the diagnostic workup. In this case, a repeat procedure may be needed to procure additional material for molecular analysis. With the increased interest in histochemical and genetic diagnosis, FNB has emerged as a viable alternative. In addition, some malignancies, such as neuroendocrine and stromal tumors, IgG4-associated disorders, and pancreatic lymphomas, often require more tissue material to allow for a definitive diagnosis [31,32]. 

As previously mentioned, the existing literature has conflicting evidence about FNA vs. FNB in terms of diagnosing PDAC. Alatawi et al. reported that the sensitivity for malignancy in FNB was 97.8% compared to 88.4% in FNA [16]. Sur et al. concluded that the diagnostic yield of FNB was 86.11% vs. 65.71% for FNA. It should be noted that FNB and FNA have shown similar diagnostic power in several studies [33,34]. Eusebi et al. found that combining both techniques improved the overall sensitivity [35]. On the other hand, Strand et al. and Yan et al. concluded in their studies that FNA had better diagnostic capabilities than FNB [11,36]. It should be noted that there were technical difficulties with FNB in the former study, which may have resulted in inadequate samples and an overall reduced FNB accuracy. A meta-analysis conducted by Renelus et al. on 11 studies, including 1365 patients, showed that FNA was associated with reduced cytopathological accuracy compared to FNB (82% vs. 89%, *p* = 0.04) [37]. In our study, both EUS-TA modalities reached similar diagnostic accuracy for attaining pathological diagnosis. This aligns with the findings from a meta-analysis, which indicated the non-superiority of 22G FNB over 22G FNA in terms of diagnostic accuracy (RR 1.02, 0.97–1.08; *p* = 0.46), sample adequacy (RR 1.01, 0.96–1.06; *p* = 0.61), and histological core procurement (RR 1.01, 0.89–1.15; *p* = 0.86) [38].

With the evolving field of personalized medicine strategies, the molecular profiling of malignancies is crucial to guide the development of treatment plans. Two white papers have emphasized the need to investigate EUS-TA methods for optimal pancreatic cancer tissue characterization and DNA extraction, as the relevance of molecular tissue analysis to clinical care has increased [39,40]. Elhanafi et al. [15] reported that EUS-FNB was more likely to yield sufficient tissue sampling for genomic testing compared to EUS-FNA in patients with PDAC (90.9% vs. 66.9%, *p* = 0.02). Our study found that FNB was more likely to result in sufficient tissue sampling for genomic and molecular testing. We demonstrated that sample adequacy for molecular testing was achieved in 71.4% of patients who underwent FNB compared to 32.1% of patients who underwent FNA (*p* < 0.001). In addition, FNB was more likely to yield samples sufficient for genomic testing compared to FNA (46.4% vs. 32.1%, *p* = 0.005). This is consistent with the studies conducted by Asokkumar et al. [41] and Dwyer et al. [42], which demonstrated the superiority of FNB compared to FNA in tissue adequacy for genomic testing. Furthermore, findings from randomized crossover trials have demonstrated a notable superiority in terms of adequacy for genomic profiling, DNA yield, and histology yield when utilizing an EUS-FNB needle in comparison to an EUS-FNA needle [43]. 

Several studies concluded that FNB biopsies were associated with a lower number of passes compared to FNA [33,44,45]. However, our study showed no significant difference in the mean number of passes needed to obtain an adequate sample (2.58 for FNB and 2.49 for FNA, *p* = 0.606). This discrepancy in results could be attributed to local expertise, considering that EUS was performed by only one endoscopist in this study, eliminating intra-observer variability. The most significant tumor characteristic in this study was tumor surface area, with a median of 25 mm^2^ for FNB vs. 4 mm^2^ for FNA (*p* < 0.001). In addition, tumor cellularity ranged from 20% to 49% in 51.0% of patients in the FNB group compared to 39.5% of patients in the FNA group (*p* = 0.079). The larger tumor surface area and cellularity obtained using FNB explain the larger sufficient tissue samples for genomic testing compared to FNA. 

Nationwide, the rate of complications (infection, hemorrhage, pancreatitis, biliary peritonitis, and celiac plexus blockade/neurolysis) from EUS is approximately 1–2% [46]. There were no reported complications following any of the procedures performed during the 4-year period in this study.

The increasing prevalence of targeted therapies necessitates a shift from the traditional approaches that we are familiar with [47]. There is a compelling requirement to explore innovative tools that can seamlessly incorporate technological advancements into our practices. This is crucial as these advancements have the potential to enhance the efficiency and precision of our diagnostic and treatment methodologies [48]. Identifying and implementing novel workflows for the management of pathological specimens is imperative. Equally important is the enhancement of communication channels facilitating the swift exchange of critical laboratory information between clinicians. These initiatives are essential for ensuring optimal and timely patient care [49,50]. 

This study has several strengths, including a relatively large and reasonable sample size compared to previous studies comparing FNA and FNB techniques. In addition, the total number of individuals who underwent genomic and molecular testing of their tumor for clinical purposes was relatively higher than in other studies (43.6% and 32.9% underwent molecular testing and genomic testing, respectively). Furthermore, all EUS procedures in our study were performed by a single endoscopist, eliminating the possibility of biased results due to differing endoscopist expertise. Additionally, genomic and molecular testing was performed for all the cases where tissue sampling was sufficient, not based on a request, making the two groups more comparable. All tissue material, whether acquired via FNB or FNA, was handled and processed similarly, limiting the bias introduced by incorporating different DNA extraction methods. Furthermore, ROSE was performed for all the cases, and there were no significant differences in the number of passes, number of swabs, tumor size, and location between the two modalities, suggesting that the sampling approaches were similar. The observation from this study was consistent with previous studies, with FNB being superior to FNA in attaining tissue adequacy for genomic testing. Notably, we did not delve into the examination of various gene panels, molecular tests, or techniques, as these aspects were beyond the scope of our research objectives. Our emphasis was specifically on examining the percentage of viable tumor and the surface area of the tumor for each specimen as an indicator of the amount of DNA collected that ensures specimen adequacy for performing the genomic tests.

We recognize several limitations in this study. First, the nature of the study as a retrospective, single-center study might limit the accurate extraction of some variables. Second, it is crucial to note that our study incorporated a relatively small cohort, potentially introducing bias in the interpretation of the results. Therefore, caution should be exercised when drawing definitive conclusions from our findings. Further studies with larger sample sizes are warranted to validate our findings. Third, most pancreatic lesions in our study were larger than 2 cm^2^ so no general conclusion can be drawn regarding smaller pancreatic lesions. Fourth, our study was conducted at a single tertiary referral center and by a single endoscopist, so the generalizability of the results is limited. Although the study included a large sample, the number of people who underwent FNB was smaller than the number of people who underwent FNA. FNA is the more commonly used sampling technique in clinical practice because it is cheaper than FNB. In addition, we used standard suction in our study. A contrast-enhanced fine-needle aspiration (CH-EUS-FNA) seems to be superior to standard EUS-FNA for patients with pancreatic masses [51]. Additionally, the use of modified wet suction appeared to yield high rates of integrity and adequate samples, albeit with increased blood contamination compared to both dry suction and no suction techniques [52]. Finally, the calculation of tumor surface area in our study involved measuring the surface area in millimeters directly from the microscopic slides. It is important to note that this method is acknowledged as somewhat crude, recognizing its limitations in precision and intricacies compared to more sophisticated measurement techniques.

## 5. Conclusions

Although there was no significant difference in the number of passes required to establish a diagnosis, EUS-FNB, guided by rapid on-site sample evaluation, was superior to EUS-FNA in obtaining adequate samples for molecular and genomic testing for PDAC. Tumor surface area and tumor cellularity were used as a guide to determine specimen adequacy, regardless of the tissue acquisition modality. 

## Figures and Tables

**Table 1:** Baseline patient, procedure, and lesion characteristics for the two modalities (FNA vs. FNB).

Characteristic	FNB (*n* = 48)	FNA (*n* = 76)	FNA and FNB (*n* = 8)
Mean age, years (±SD)	69.88 ± 10.38	72.11 ± 10.86	67.25 ± 9.44
Male, n (%)	22 (45.8%)	36 (47.4%)	6 (75.0%)
CEA level, n Median (Q1, Q3)	15 5.40 (2.70, 47.90)	27 5.10 (1.70, 16.90)	2 8.70 (6.60, 10.80)
CA 19-9 level, n Median (Q1, Q3)	35 952.00 (129.00, 4483.00)	53 312.00 (43.00, 1661.00)	5 195.00 (174.00, 223.00)
Complications, n (%)	0	0	0
	**FNB (*n* = 56)**	**FNA (*n* = 84)**	***p-*** **value**
Pancreatic lesion size in cm^2^			0.713
< 2 cm^2^	4 (7.1%)	4 (4.8%)	
≥ 2 cm^2^	52 (92.9%)	80 (95.2%)	
Pancreatic lesion location, *n* (%)			0.719
Body	7 (12.5%)	11 (13.1%)	
Body and tail	7 (12.5%)	5 (6.0%)	
Head/uncinate process	28 (50.0%)	50 (59.5%)	
Head and neck	5 (8.9%)	4 (4.8%)	
Neck and body	1 (1.8%)	3 (3.6%)	
Neck/genu	2 (3.6%)	2 (2.4%)	
Tail	6 (10.7%)	9 (10.7%)	
Needle gauge, *n* (%) *			< 0.001
19 G	22 (40.0%)	10 (11.9%)	
22 G	32 (58.2%)	69 (82.1%)	
25 G	1 (1.8%)	5 (6.0%)	

Categorical variables compared using Chi-square or Fisher’s exact test; *: There is one data point missing from the FNB group.

**Table 2:** Comparison of outcomes for FNA vs. FNB.

Outcome Measure	FNB (*n* = 56)	FNA (*n* = 84)	*p*-Value *
Mean pass counts (±SD) **	2.58 ± 1.06	2.49 ± 1.07	0.5096
Sample adequacy for molecular testing, *n* (%)	35 (71.4%)	26 (32.1%)	< 0.001
Genomic testing performed, *n* (%)	26 (46.4%)	20 (23.8%)	0.005

* Outcomes were statistically analyzed after adjusting for age, sex, pancreatic lesion location, and size. ** There are three missing from the FNB group.

**Table 3:** Comparison of the pathological characteristics using FNA vs. FNB.

Characteristic	FNB (*n* = 56)	FNA (*n* = 84)	*p*-Value
Tumor Surface Area in mm^2^, *n* Median (Q1, Q3)	49 25.00 (4.00, 100.00)	81 4.00 (1.00, 25.00)	<0.001
Tumor cellularity * <20% 20–49% >49%	9 (18.4%) 25 (51.0%) 15 (30.6%)	30 (37.0%) 32 (39.5%) 19 (23.5%)	0.079
Mean number of smear slides (±SD)	4.30 ± 2.82	4.49 ± 2.80	0.704

Continuous variables were compared using Student’s T-tests or Wilcoxon rank-sum tests. Categorical variables were compared using Chi-square or Fisher’s exact tests. * There are seven data points missing from the FNB group and three missing from the FNA group.

## Data Availability

Data are available upon request from the corresponding author.

## Abbreviations

PDAC	Pancreatic Ductal Adenocarcinoma
CT	Computerized Tomography
EUS	Endoscopic Ultrasound
MRI	Magnetic Resonance Imaging
EUS-TA	Endoscopic Ultrasound Tissue Acquisition
FNA	Fine-Needle Aspiration
FNB	Fine-Needle Biopsy
CNB	Core Needle Biopsy
EUS-FNA	Endoscopic Ultrasound Fine-Needle Aspiration
EUS-FNB	Endoscopic Ultrasound Fine-Needle Biopsy
KC	Kansas City
MO	Missouri
CA	Cancer Antigen
CEA	Carcino-Embryonic Antigen
ROSE	Rapid On-site Evaluation
RPMI	Roswell Park Memorial Institute
Q1, Q3	Quartile 1, Quartile 3
IgG 4	Immunoglobin G 4
